# Heavy metal uptake by plant parts of *Populus* species: a meta-analysis

**DOI:** 10.1007/s11356-023-27244-2

**Published:** 2023-05-03

**Authors:** Dávid Tőzsér, Roland Horváth, Edina Simon, Tibor Magura

**Affiliations:** 1 Department of Ecology, University of Debrecen, Egyetem sq. 1, Debrecen, H-4032 Hungary; 2 Circular Economy Analysis Center, Hungarian University of Agriculture and Life Sciences, Páter Károly str. 1, Gödöllő, H-2100 Hungary; 3 ELKH-DE Anthropocene Ecology Research Group, University of Debrecen, Egyetem sq. 1, Debrecen, H-4032 Hungary

**Keywords:** Poplar, Phytoremediation, Effect size, Accumulation, Pollution, Literature search

## Abstract

*Populus* species are well documented for being potentially suitable for phytoremediation purposes regarding their accumulation characteristics. However, published results are contradictory. Based on the data gathered during an extensive literature search, we aimed to assess and revise the metal accumulation potential in the root, stem, and leaf of *Populus* species growing in contaminated soils, with meta-analysis. We evaluated the influences of pollution level, soil pH, and exposure time on the metal uptake patterns. We found accumulations of Cd, Cr, Cu, Pb, and Zn to be significant in each plant part, while that was only moderate for Ni, and limited for Mn. By calculating the soil pollution index (PI), we observed significantly intensive, PI-independent accumulation for Cd, Cr, Cu, Ni, Pb, and Zn. A decrease in soil pH significantly increased the uptake of Mn and significantly decreased the accumulation of Pb in the stem. Metal uptake was significantly influenced by exposure time as well; Cd concentration was significantly decreased in the stem, while concentrations of Cr in the stem and leaf, and Mn in the stem were significantly increased with time. These aforementioned findings support a well-founded metal-and-growth condition-specific application of poplars in phytoremediation processes, also triggering further in-depth assessments to enhance the efficiency of relevant poplar-based technologies.

**Supplementary Information:**

The online version contains supplementary material available at 10.1007/s11356-023-27244-2.

## Introduction

Soil pollution and contamination pose a high risk limited not only to the soil itself but also to all the associated compartments and compounds in its environment (Briggs 2003; Skála et al. 2018). The complex and, in many instances, unknown effect mechanism of certain contamination cases has recently been accompanied by the gradually increasing diversity of compounds emitted into our environment, which then turns into an even bigger challenge to face (Delang 2017; Lemmel et al. 2019). Therefore, the nature of contamination urges the need for joint action from stakeholders.

Among chemical substances, metals are widely studied in environmental assessments (Wei et al. 2021). It is known that metal contamination directly affects the lives of tens of millions of people all around the world, while this number is even higher if we consider people indirectly concerned (Shakoor et al. 2013; Zhai et al. 2018). Metals are persistent and non-degradable, which are both critical regarding their fate; in addition, the density of metals highly determines all the basic characteristics of these elements and their compounds (Khalid et al. 2016). Among metals, heavy metals constitute a broad group. The definition and determination of these elements and their compounds are, however, subject to universal debate; according to one of the major consensuses, an excess amount of these substances in the environment is generally harmful (Ali and Khan 2018; Aliyev et al. 2018). This latter finding corroborates the need for thorough assessments of metal contamination.

It has previously been revealed that the prevention of soil contamination is much more desirable than its elimination both from an ecological and economical point of view (Ram et al. 2013). In case the contamination cannot be or is not intended to be avoided, there are still viable options to mitigate the harmful effects of a contaminant in an environmentally sound and economical manner (Jeelani et al. 2017). Phytoremediation, as a continuously developing group of phytotechnologies, exploits the inherent and/or intensified potential of plant species to grow under various conditions, while showing ability to accumulate, stabilize, degrade, and/or transform a wide range of substances (Lee 2013; Cristaldi et al. 2017; Parihar et al. 2021). In contrast with conventional remediation methods, phytoremediation operates with only negligible disturbance caused in its environment offering a mid-to-long-term green cover in the affected area (Ali et al. 2013).

One of the major aspects of plant-based remediation is the accumulation of contaminants (e.g., metals) in tissues, called phytoextraction. The principle behind the mechanism builds on the fact that certain plants can accumulate metals via roots and store a great proportion of those in underground plant parts with contaminant- and species-dependent tendencies to translocate them into aboveground parts (Suman et al. 2018; Antoniadis et al. 2021; Castañeda-Espinoza et al. 2022). To make phytoextraction effective and the whole process economical, selected plant species must represent specific characteristics including rapid growth (biomass production) and a high degree of metal tolerance (Arthur et al. 2005; Shikha and Singh 2021). For these reasons, the best candidates can be found among woody species (Mohsin et al. 2019; Simon et al. 2022). In addition to the abovementioned characteristics, the efficiency of remediation can be facilitated by genetic modification (e.g., overexpression of genes accountable for metal tolerance and uptake) or by increasing the bioavailability of metals in the growing media (Liu et al. 2020; Sarma et al. 2021; Trippe and Pilon-Smits 2021). Earlier papers published beneficial information on the metal accumulation potential of members of the Salicaceae family, out of which *Populus* (poplar) is a thoroughly mapped genus in this regard (Guerra et al. 2011; Marmiroli et al. 2011). The advantages of this genus over other genera with rapid growth and high biomass production rate (e.g., *Brassica* and *Sorghum* species) are its high biomass, soil stabilization effect due to the deep root system, continuous metal accumulation, and applicability in short rotation coppice (SRC) management even in the long term at the same time, favoring its common use for phytoremediation purposes (Chandra et al. 2016; Villette et al. 2019).

Along with the frequent application of poplars, inconsistencies can be discovered among the published results on metal accumulation in plant parts. For instance, Pietrini et al. (2010) studied the Cd accumulation in 10 *Populus* clones. The authors observed great variability in leaf accumulation among clones, with concentrations remaining generally low. In contrast, Langer et al. (2009) found that leaves were the main metal depositories in *Populus × canescens* (Aiton) Sm. A variable interspecific uptake pattern was found by Borghi et al. (2008) who demonstrated different responses of poplar species to similar Cu supply. Earlier papers also indicated that differences in metal uptake in organisms can be traced back to variations in pollution levels of their habitats. Adams et al. (2011) presented major differences in metal accumulation of *Populus trichocarpa* Torr. & A.Gray ex Hook. growing in environments with different levels of Zn contamination. Studying differently contaminated sites, Algreen et al. (2014) found a positive correlation between plant and soil Cd and Ni concentrations, while results for Cu and Zn were contradictory. A great number of studies indicated that soil acidity (pH) is highly relevant in determining the bioavailability, thus the intensity of accumulation in woody species. Mleczek et al. (2009) found a negative correlation between soil pH and metal uptake in *Salix viminalis* L. individuals. The same phenomenon was presented by Hu et al. (2019) for Ni in the case of *Populus hopeiensis* Hu & H.F.Chow. Additionally, the length of time that plants are exposed to the heavy metal may also be an important determining factor of accumulation. Di Baccio et al. (2009) highlighted for *Populus × canadensis* Moench (also known as *Populus euramericana* (Dode) Guinier) that the species showed a highly exposure time-dependent Zn accumulation. However, a positive correlation between time of exposure and tissue metal enrichment was reported to be non-significant in woody species after reaching certain concentrations (Tőzsér et al. 2017). In conclusion, the evaluation of these factors can be valid in studies to get a comprehensive view of selected species.

This study aimed to investigate and consolidate the published data on metal uptake among the selected plant parts (root, stem, and leaf) of *Populus* spp. grown in contaminated soils with tools of meta-analysis. We selected studied metals by their environmental relevancy and data availability; therefore, accumulations of cadmium (Cd), chromium (Cr), copper (Cu), manganese (Mn), nickel (Ni), lead (Pb), and zinc (Zn) were assessed. In this paper, we studied, how and to what degree (I) *Populus* spp. accumulate metals in plant parts; (II) metal uptake is correlated with the pollution intensity level of soil; (III) plants accumulate metals under acidic and alkaline soil conditions; (IV) metal concentrations are correlated with exposure time in plant parts.

## Materials and methods

### Literature search and data selection

We collected the data through a literature search using all the databases available on the Web of Science for the period 1975–2021. On this platform, we used the following terms: TOPIC = (*Populus*) AND TOPIC = (metal OR phytoremediation). To have an extensive overview of the publications being appropriate for the analyses we also reviewed the reference section of each paper. We considered a publication appropriate for the meta-analysis in case it reported the mean concentrations (in mg kg^-1^, referred to dry matter) with their variances and sample sizes for one or more of the most relevant metals (Cd, Cr, Cu, Mn, Ni, Pb, Zn) in plant parts (root and/or stem and/or leaf) and in soil from both contaminated and uncontaminated (control) areas. Papers assessing the effects of accumulation-stimulative compounds on the accumulation pattern of plants were excluded from the analyses. We retrieved the required data from text, tables, and figures.

### Statistical analyses

We calculated unbiased standardized mean difference (Hedges’ *g*) for the meta-analyses, as a common effect size of the contaminated-uncontaminated comparisons.

1 $$g=J\frac{{\overline{x}}_U-{\overline{x}}_C}{S_{within}}$$

2 $${S}_{within}=\sqrt{\frac{\left({n}_U-1\right){S}_U^2+\left({n}_C-1\right){S}_C^2}{n_U+{n}_C-2}}$$

and

3 $$J=1-\frac{3}{4\left({n}_U+{n}_C-2\right)-1}$$

where $\overline{X_U}$ and $\overline{X_C}$ are the mean metal concentration (mg kg^-1^) in plant parts of poplars growing in uncontaminated (U) and contaminated (C) soils, $n_{U}$ and $n_{C}$ are the sample sizes for poplar parts growing in uncontaminated (U) and contaminated (C) soils, $S_{U}$ and $S_{C}$  and are the standard deviations. Negative *g* values refer to a higher metal concentration in plant parts of poplars from contaminated soils than individuals from uncontaminated ones. We used subgroup meta-analysis to assess the similarity in metal accumulation from contaminated soils among the different plant parts of the poplar species; the three subgroups were root, stem, and leaf.

We performed a random-effects model to estimate the overall effect and the effects of moderators (plant parts of poplars). Generally, there is a high variation in locations, conditions, experimental setups, and methods used in the individual studies, which did not enable us to estimate a common effect size; thus, we used a random-effects model (Borenstein et al., 2009). Random-effect models have a higher degree of plausibility than fixed-effect ones; they attribute the distribution of effect sizes to real differences among studies and do not assume sampling error as the only source of differences in effect sizes (Borenstein et al., 2009). As more than a single effect size was computed from one study, we included a publication-level random effect as a nesting factor in the random-effect models. We considered the mean effect size statistically significant if the 95% bootstrap confidence interval (CI; calculated with 999 iterations) did not include zero.

We also assessed effect size homogeneity across studies; in the case of variable effect sizes, the interpretation of results would be substantially different than in the case of consistent ones. We also calculated complementary measures of heterogeneity (*Q*, *T*^*2*^, and *I*^*2*^) to describe the heterogeneity of effects between studies (Borenstein et al., 2009). We partitioned total variance (*Q*_*total*_) into within-groups (*Q*_*within*_) and between-groups (*Q*_*between*_) variances using a *Q* test based on the analysis of variance; we tested these different components of variance for statistical significance (Borenstein et al., 2009). We considered variance between groups (*Q*_*between*_) significant if metal accumulation from contaminated soils was significantly different (*p*<0.05) among plant parts of poplars. Additionally, we evaluated the proportion of true variance explained by covariates (subgroup classification) by the calculation of *R*^*2*^ (Borenstein et al., 2009). We excluded subgroups with less than four cases from the analyses during calculations.

In meta-analyses, publication bias resulting in missing studies and biased effect sizes are also common. We used funnel plots and Egger’s test to assess publication bias (Borenstein et al., 2009). In addition, we used the trim and fill method in case of significant asymmetry (Duval and Tweedie, 2000). The trim and fill method calculates the number of missing studies, while their effect sizes and standard errors are also computed. Missing studies are then added to the dataset of the meta-analysis, followed by the re-computation of the summary effect size. Using this method, an unbiased estimate of the summary effect size is given (Borenstein et al. 2009).

We performed subgroup meta-analyses to investigate whether metal accumulation was different among species from soils with different levels of pollution. The subgroups were the soils with different pollution intensity levels. We studied the level of pollution by calculating the pollution index (PI); it represents the ratio of the measured and the background soil metal concentration (mg kg^-1^) (Faiz et al. 2009):

4 $$PI=\frac{M_c}{B_c}$$

where *M*_*c*_ is soil metal concentration presented by selected papers; *B*_*c*_ is background soil metal concentration. According to the equation, the following pollution intensity level groups were set: PI ≤ 1 (low), 1 ≤ PI ≤ 2 (moderate), 2 ≤ PI ≤ 5 (high), and PI ≥ 5 (extreme) (Lu et al. 2007; Simon et al. 2013; Tőzsér et al. 2019). We extracted reference background metal concentrations from the Geochemical Atlas of Europe Part 1 (Salminen et al. 2006).

We performed each of the meta-analyses, heterogeneity measures, and assessment of publication bias using the *MAd* and *metafor* packages (Viechtbauer 2010; Del Re and Hoyt 2014) operated in the R (version 4.1.2; R Core Team 2018). To assess soil acidity-dependent and temporal metal accumulation in plant parts of poplars, we analyzed the relationship between Hedges’ *g* (standardized mean difference) and soil pH, and between Hedges’ *g* and exposure time, respectively. We assessed the relationships by linear models using the *lm* method, carried out in the R.

## Results

### Literature search

The literature search provided 15,347 publications. Out of these, 29 papers presented results on the mean concentrations of metals (Cd, Cr, Cu, Mn, Ni, Pb, and/or Zn) with their variances and sample sizes from soils and plants, in case of both contaminated and uncontaminated (control) sites (Table 1, Supplementary Materials A). Assessing the selected publications, a total number of 528 comparisons could be extracted. The level of contamination in soils was very wide-ranging in case each of the studied metals (0.12–150 mg kg^-1^ for Cd, 20.8–13,600 mg kg^-1^ for Cr, 14.4–2396 mg kg^-1^ for Cu, 287–2790 mg kg^-1^ for Mn, 13.2–111 mg kg^-1^ for Ni, 7.0–970 mg kg^-1^ for Pb, and 49.1–6013 mg kg^-1^ for Zn. In the selected publications, 11 *Populus* species, clones, and hybrids were studied (Table 1). As a preliminary assessment, we found metal accumulation trends in plant parts similar both when analyzing the species separately and together (results are not shown); thus, in further analyses, we assessed *Populus* species together.

**Table 1:** Publications used in the meta-analysis, which reported the mean values of the metal concentrations, standard deviations, and sample sizes for at least one species growing on contaminated and uncontaminated soils

Paper	Studied species/hybrid(s)	Studied plant part(s)	Studied heavy metal(s)	Number of comparisons
Alizadeh et al. 2012	*Populus alba*	Leaf, root	Cd	6
Castiglione et al. 2009	*P. alba, P. nigra*	Leaf, stem, root	Cu, Zn	18
Ciadamidaro et al. 2013	*P. alba*	Leaf	Cd, Cu, Mn, Pb, Zn	50
Ciadamidaro et al. 2014	*P. alba*	Leaf	Cd, Cu, Mn, Pb, Zn	10
Cicatelli et al. 2010	*P. alba*	Leaf, stem, root	Cu, Zn	8
Dos Santos Utmazian & Wenzel 2007	*P. tremula, P. nigra*	Leaf, root	Cd, Zn	8
Durand et al. 2010	*P. tremula × alba*	Leaf, stem, root	Zn	3
Durand et al. 2011	*P. tremula × alba*	Leaf, stem, root	Cd, Zn	4
El-Mahrouk et al. 2020	*P. nigra*	Leaf, stem, root	Cd, Cu, Pb	36
Evangelou et al. 2013	*P. nigra ‘Monviso’*	Leaf, stem	Cd, Cu, Mn, Zn	35
Guo et al. 2015	*P. euramericana*	Leaf, stem, root	Cd	6
Günthardt-Goerg et al. 2019	*P. tremula*	Leaf, stem, root	Cd, Cu, Pb, Zn	56
Hassinen et al. 2009	*P. tremula × tremuloides*	Leaf, root	Cd, Cr, Cu, Ni, Zn	20
Hermle et al. 2007	*P. tremula*	Leaf	Cd, Zn	16
Hu et al. 2013a	*P. alba*	Leaf, stem, root	Cd, Cu, Pb, Zn	60
Hu et al. 2013b	*P. alba*	Leaf, stem, root	Cd	15
Lettens et al. 2011	*P. trichocarpa × deltoides*	Leaf	Cd, Cu, Mn, Zn	7
Lingua et al. 2008	*P. alba, P. nigra*	Leaf, stem, root	Zn	6
Lingua et al. 2012	*P. alba*	Leaf, stem, root	Cu, Zn	10
Madejón et al. 2004	*P. alba*	Leaf, stem	Cd, Cu, Mn, Ni, Pb, Zn	12
Mandre 2014	*P. tremula × tremuloides*	Leaf, stem	Cd, Cr, Cu, Mn, Pb, Zn	12
Nikolić et al. 2017	*P. euramerica, P. deltoides*	Leaf, stem, root	Cd	6
Pilipović et al. 2019	*P. deltoides*	Leaf, root	Cd, Cr, Cu, Ni, Pb, Zn	36
Rafati et al. 2011	*P. alba*	Leaf, stem, root	Cd, Cr, Ni	27
Sebastiani et al. 2004	*P. euramericana,* *P. deltoides × maximowiczii*	Leaf, stem, root	Cr, Zn	12
Stobrawa & Lorenc-Plucińska 2008	*P. nigra*	Root	Cd, Cr, Cu, Mn, Ni, Pb, Zn	7
Szuba & Lorenc-Plucińska 2017	*P. alba*	Leaf, root	Cr, Cu, Pb, Zn	8
Vandecasteele et al. 2003	*P. trichocarpa × deltoides*	Leaf	Cd, Zn	22
Wu et al. 2010	*P. deltoides × nigra*	Leaf, root	Cd	12

### Accumulation of metals in *Populus* tissues

**Fig. 1:**
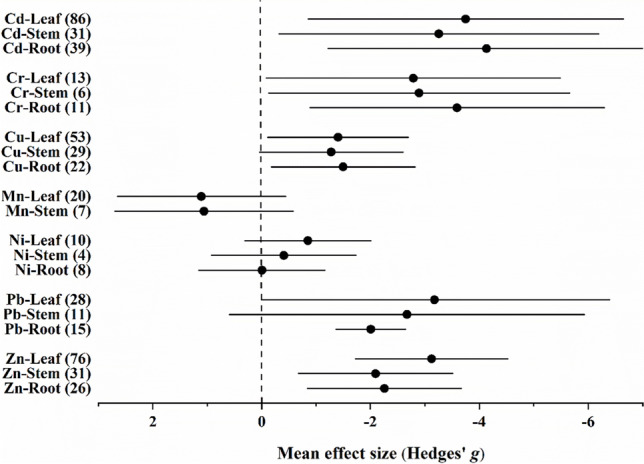
Mean effect sizes of random-effect models (mean Hedges’ *g* ± 95% confidence interval) for Cd, Cr, Cu, Mn, Ni, Pb, and Zn accumulations in *Populus* plant parts. Values in brackets refer to the number of comparisons from which the mean effect size was calculated. A negative *g* value means higher metal concentration in plant parts of *Populus* spp. growing on contaminated sites than on uncontaminated ones. The mean effect size was considered statistically significant if the 95% bootstrap confidence interval (CI) did not include zero

Accumulation was significantly higher in all plant parts (root, stem, and leaf) for Cd, Cr, and Zn, compared to control individuals. It was significantly higher in the root and leaf, but not in the stem for Cu and Pb. The accumulation in all plant parts for Ni was higher, while that for Mn was lower in individuals growing in contaminated soils compared to ones growing in uncontaminated soils, although these differences were not significant (Fig. 1 and Supplementary Materials B1–B7). We found differences 2790 in the accumulation of Cr, Pb, and Zn among plant parts (Supplementary Materials B1–B7). In the overall model, total heterogeneity was significant for each of the studied metals, while significant residual, unexplained heterogeneity was also found (Supplementary Materials B1–B7). In the funnel plot, both (classical and random-effects) versions of Egger’s test indicated significant asymmetry for Cd, Cr, Cu, Ni, Pb, and Zn, while for Mn the classical version did not indicate significant asymmetry; however, the random-effects version did (Supplementary Materials C1–C7). Further, the number of missing values estimated by the trim and fill method was 36 for Cd, 34 for Zn, 19 for Pb, 13 for Cu, 5 for Ni, and 0 for Cr and Mn (Supplementary Materials C1–C7). Adding the detected missing data points, however, did not change the outcome of the models (Supplementary Materials C1–C7).

### Correlation between soil pollution index (PI) and metal accumulation in *Populus* tissues

Accumulation patterns of plant parts under different pollution intensity levels could be assessed for Cd in the leaf, Cr in the root, and Zn in the stem and leaf, having a sample size of *n*≥4. During the analysis, we found no significant relationship between the accumulation rate in plant tissues and the pollution intensity (Supplementary Materials D).

### Correlation between soil pH and metal accumulation in *Populus* tissues

Studying the metal accumulation in plant parts of *Populus* spp. under different acidity conditions, we found one significant and one marginally significant correlation (Supplementary Materials E1–E7). Accumulation of Mn in leaves was marginally significantly decreased with the increase of soil pH (*F*=4.32, *n*=20, *p*=0.052, *R*=0.44); it means that the difference in leaf metal concentrations decreased between individuals from contaminated sites and uncontaminated ones with increasing soil pH.

**Fig. 2:**
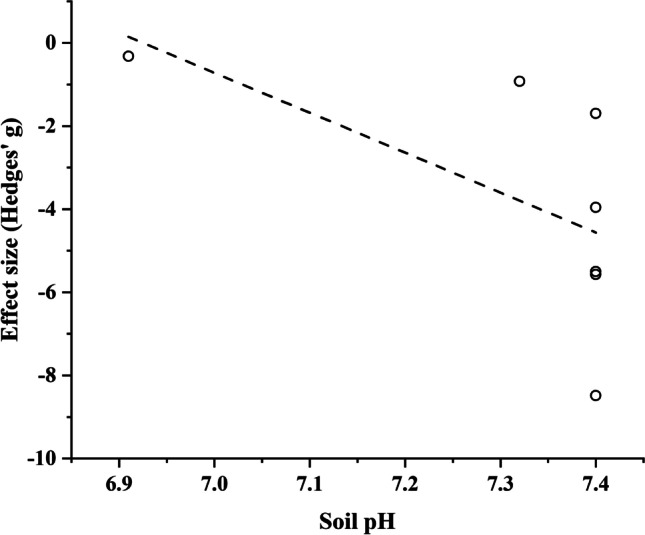
The relationship between the standardized mean difference (Hedges’ *g*) calculated for Pb concentrations in stems of *Populus* spp. growing on contaminated vs. uncontaminated sites and soil pH. More negative *g* values indicate a higher difference in Pb concentration between the stems of *Populus* spp. growing on contaminated vs. uncontaminated sites (*F*=6.4, *n*=11, *p*=0.033, *R*=−0.64)

Contrarily, accumulation rates of Pb in stems were significantly increased with the increase of soil pH (Fig. 2), which means that the difference in stem metal concentrations significantly increased between individuals from contaminated sites and uncontaminated ones with increasing soil pH.

### Temporal accumulation of metals in Populus tissues

To study the time-dependent accumulation of the studied metals in *Populus* spp. tissues, we found four significant correlations (Table 2; for the other relationships see Supplementary Materials F1–F7). The rate of Cd accumulation was significantly decreased with exposure time in stems of *Populus* spp., suggesting a significantly decreasing difference with time in Cd concentrations of stems between *Populus* spp. from contaminated vs. uncontaminated sites.

**Table 2:** The significant relationship between the standardized mean difference (Hedges’ *g*) calculated for metal concentrations in tissues of *Populus* growing on contaminated vs. uncontaminated sites and the exposure time

Component of variance	*d.f.*	Sum of squares	Mean square	*F* value	*p* value
*Cd accumulation in stem*					
Model	1	1601.331	1601.331	5.750	0.023
Error	29	8076.539	278.501		
Total	30	9677.871			
*Cr accumulation in stem*					
Model	1	100.038	100.038	52.057	0.002
Error	4	7.687	1.922		
Total	5	107.725			
*Cr accumulation in leaf*					
Model	1	62.316	62.316	54.852	<0.001
Error	11	12.497	1.136		
Total	12	74.813			
*Mn accumulation in stem*					
Model	1	649.017	649.017	674.676	<0.001
Error	5	4.810	0.962		
Total	6	653.827			

In contrast, the accumulation of Cr in stems and leaves, and Mn in stems of *Populus* spp. were significantly increased with exposure time (Table 2); it indicated that a strong increase could be expected in the accumulation intensity of the metals in *Populus* plant parts as the exposure got longer.

## Discussion

### Accumulation of metals in *Populus* tissues

We identified that among the seven studied metals Cd, Cr, and Zn were accumulated in *Populus* spp. in significantly higher concentrations from contaminated soils than in individuals from uncontaminated ones. However, accumulation among plant parts varied according to the heavy metal. For Cu, Ni, and Pb, differences were non-significant in some cases and significant in others. The accumulation intensity of Mn was lower in individuals growing on contaminated soils than in individuals on uncontaminated ones. Reading the earlier results of phytoremediation studies, the results about the accumulation patterns of metals in *Populus* spp. are contradictory.

We demonstrated using meta-analysis that the root, stem, and leaf of *Populus* spp. accumulated Cd in similarly high concentrations; thus, we did not find significant differences among the plant parts. There is variation among published results regarding the level of accumulation and the compartmentation of Cd in plants. Besides highlighting the overall great accumulation potential of the species, Baldantoni et al. (2014) found significantly higher Cd concentrations in leaves than in stems and roots of *Populus nigra* L. In cases of three *Populus* spp. clones Ruttens et al. (2011) also confirmed the Cd accumulation primarily in leaves; the phenomenon was linked to the good translocation potential of Cd in *Populus* spp. and the simultaneous large bioavailable quantity of the metal in the studied low-nutrient soil. On the contrary, growing four poplar hybrids in a media with elevated (100–500 mg l^-1^) Cd supply, Chandra et al. (2016) found the concentration much higher in roots than in leaves and stems. Shim et al. (2013) also identified root compartmentation of Cd in transgenic poplars, explaining the results by the increased Cd-storing capacity of the more extensive root system compared to genetically non-modified species. Among all metal-plant part relations, we found the accumulation of Cd to be the most intensive in roots, followed by the leaves of poplars; in particular, a great proportion of the relevant data was presented for *Populus alba* L., *P. nigra*, and their relative hybrids, which suggests outstanding Cd accumulation performance not only for the genus in general but also for these specified species and hybrids. Intensive root accumulation could thus be attributed to the wide species-dependent variation of poplars in metal uptake and transport, based on the strategy developed to avoid aboveground parts being impaired under metal stress (Iori et al. 2016). According to Pajević et al. (2009), the role of metal interactions in the soil determines the Cd accumulation in poplars; the authors found multi-contamination to cause condition drop-based accumulation discrepancies in *Populus deltoides* W.Bartram ex Marshall and *P. euramericana*. Dos Santos Utmazian et al. (2007) also listed, besides other factors, the elemental composition of soils as a major force influencing the uptake of Cd in poplars. In this analysis, papers involving genetically modified poplars and soils with different levels of metal contamination were also included, thus the (mutual) weight of these factors in forming the accumulation pattern should not be excluded. Nonetheless, we found only minor differences in Cd accumulation among plant parts, which supports the conscious handling of the whole individuals during and after the remediation of Cd-affected soils.

Similar to the result for Cd, we did not indicate significant differences in Cr accumulation among plant parts of poplars, while the level of uptake was significant in each case as well. Studying the phytoremediation potential of *P. nigra* hybrid “Monviso,” Ancona et al. (2017) reported much higher Cr concentrations in roots than in leaves; the authors supported the use of the hybrid for Cr phytostabilization instead of for phytoextraction purposes. Pulford and Watson (2003) explained the low translocation of Cr in plants by the specific physico-chemical properties of the metal, which make Cr one of the metals generally immobilized in roots and root zone. In our study, we also observed the prime role of root accumulation for *Populus* (particularly for *P. alba* and *P. deltoides*, which two species and their hybrids gave two-thirds of the total Cr-related comparisons in the analyses), while the difference was, however, less pronounced than indicated by several previous papers. In their experiment, Kacálková et al. (2014) described the studied hybrid (*Populus nigra* L. × *Populus maximowiczii* A.Henry “NM6”) as a root accumulator for Cr by moderate soil contamination (total soil concentrations: 39–141 mg kg^-1^) levels. The authors found low (<1.1 mg kg^-1^) tissue concentrations in each case, which was the result of the very low (<0.005 mg kg^-1^) bioavailable fraction in soil, which latter was a result of soil parameters such as pH and organic matter content. Low uptake and translocation rates were reported by Chandra et al. (2016) in cases of four poplar hybrids. Bing et al. (2016) also found low accumulation rates for Cr in *Populus purdomii* Rehder root; however, as a contrary finding, aboveground plant parts were identified as the main Cr depots, which reflected on a potential atmospheric source. In this study, we aimed to include papers with a sampling design characterized by a low risk of metal accumulation via atmospheric pathways, focusing on the soil uptake of metals exclusively.

Metal accumulation potential of poplars for Cu was significant in roots and leaves, and marginally significant in stems, without showing major differences among plant parts. After comparing several candidate species for phytoextraction, Antonijević et al. (2012) referred to *P. nigra* as a root accumulator for Cu with very high (170–2400 mg kg^-1^) tissue concentrations growing in moderately to heavily contaminated (440–3800 mg kg^-1^) soils. Likewise, Pietrini et al. (2017) found significantly higher concentrations in roots than in aboveground parts of *P. nigra* hybrid “Monviso,” confirming a specifically restricted translocation of Cu in poplars reported by earlier papers (Pulford and Watson 2003). Contrasting results can also be recovered from the literature. Cordero et al. (2013) observed the Cu remediation potential of *P. nigra* to be only moderate with the highest concentrations detected in leaves. Kalubi et al. (2016) came to the same conclusion studying *Populus tremuloides* Michx.; soil parameters and adaptation mechanisms of the species were mentioned as the major influences. Besides these factors, interspecific variation in metal accumulation and compartmentation was also listed as accountable for the different results under similar experimental conditions (Borghi et al. 2008) and the accumulation pattern presented in this paper.

For Mn, we found that accumulation was very restricted in leaves and stems of poplars; accumulation was hindered in species growing in contaminated soils. The low accumulation rates can be traced back to the specific traits of Mn and soils. De Santo et al. (2002) assessed the foliar Mn concentration in *Populus tremula* L. individuals and reported it to be low to moderate; this pattern was linked to the organic matter content of the soil, which, despite the relatively large mobile pool, bound Mn strongly to make the metal barely available for plants. Millaleo et al. (2010) emphasized that many plant species have well-developed defensive (avoidance) mechanisms to cope with Mn contamination (e.g., sequestration in less active compartments). As an interpretation of low Mn uptake in previous papers, Cd-antagonism (Wang et al. 2018), root retention by metal complexes, and interactions with nutrients (e.g., K and Mg) (Alam et al. 2005) have already been introduced. Further, species-specific accumulation is very characteristic of Mn (Foy et al. 1988; Millaleo et al. 2010), which was also proved by Shukla et al. (2011), who found major differences among the four studied woody species; the tissue-soil accumulation ratio for Mn of *P. alba* exceeded that for Cd, Cu, Ni, Pb, and Zn. Monitoring several plants growing in an industrial area Saba et al. (2015) concluded that *P. nigra* had the highest Mn remediation performance based on its very high leaf concentrations. Giachetti et al. (2006) also found intensive Mn translocation in poplars. In this paper, we found low translocation of Mn into aboveground plant parts, while due to the lack of data on root concentrations, we can neither support nor deny the possibility of root retention.

Based on the data available, we presented good accumulation potential for Ni in poplars in contaminated soils, but the rate of uptake was non-significant in each of the plant parts. Ni is known to be a metal intensively translocated from roots towards leaves (Pulford and Watson 2003). In line with our findings, Kalubi et al. (2016) found the highest Ni concentrations in leaves compared to roots and aboveground woody parts; however, the differences the authors reported were significant among plant parts and concerning the soil as well. Similar to our results Bing et al. (2016) observed the highest Ni concentration in the leaves of poplars, whereas uptake intensity from soil remained low. The available literature established the good translocation potential of Ni; however, the role of factors such as interspecific variation (Houda et al. 2016), metal amount, speciation, interactions, and other soil parameters (e.g., pH, Pajević et al. 2009; Hassan et al. 2019) in accumulation patterns should also be considered. Consequently, under specific soil conditions, poplars could retain Ni in roots; Kacálková et al. (2014) reported that after being exposed to a multi-contaminated environment 66% of the accumulated Ni remained in roots. We suppose that moderate (13–111 mg kg^-1^) soil Ni concentrations and elemental composition of the soils involved in this paper, and the variety of species in individual studies contributed to the overall moderate performance of poplars respecting Ni accumulation.

During the analyses, we found the accumulation of Pb to be significant in leaves and roots, while uptake was also substantial in stems of poplars. Pb is usually referred to as one of the least mobile elements, thus with very limited bioavailability (Amin et al., 2018). Previous research results on the accumulation of Pb in poplars confirmed this prior statement, but exceptions also exist. Baldantoni et al. (2014) found that *P. alba* and *P. nigra* can sequester most Pb in root vacuoles, thereby protecting the physiologically vulnerable organs from being affected by metal toxicity. In a study on Pb uptake in *P. nigra* Mrnka et al. (2012) found lower Pb concentrations in leaves than in shoots in each of the experimental designs. In accordance, Ruttens et al. (2011) reported higher Pb concentrations in the leaf than in the stem of three poplar clones. Different experimental build-ups (soil elemental compositions), however, resulted in different accumulation patterns of *P. nigra* “Monviso” in the study of Ancona et al. (2017); in the first and second sets, the authors found elevated Pb in leaves compared to roots, while the contrary was observed in the third and fourth sets, which underlined the basic role of metal interactions. In our study, roots were the primary organ of Pb accumulation, while the adaptation/avoidance mechanism was assumed to have a lowering effect on the Pb concentration in the stem and leaf.

Analyzing the data from the papers involved, we demonstrated poplars to have significant Zn accumulation potential in roots, stems, and leaves as well, with leaves having higher metal concentrations than the other two plant parts. The high degree of Zn mobility from the soil into roots and then into aboveground parts was confirmed by Todeschini et al. (2011) from single-contaminated and by Vollenweider et al. (2011) from multi-contaminated experiments, who named the leaf as the plant part of poplars with the highest remediation potential. In contrast, Benyó et al. (2016) presented low translocation rates for Zn from roots into leaves, concluding that roots of *P. deltoides* and *P. canadensis* accumulated and restrained most of the metals growing in Cu/Zn multi-contaminated media. The uptake of Zn is frequently discussed concerning the presence of other metals, mainly Cd (Tőzsér et al. 2017). The correlation between the two metals is not uniformly evaluated; some papers supposed a positive correlation (synergism) in plant accumulation between Zn and Cd (Brekken and Steinnes 2004; De Oliveira and Tibbett 2018), while others found a negative correlation (antagonism) between them (Adiloglu 2002; Podar et al. 2004; Tkalec et al. 2014). We found comparable accumulation patterns for Zn and Cd in this study; however, trends among plant parts and the degree of accumulation were different—thus, we do not suppose antagonism between the metals. Further, Xue et al. (2020) referred to short rotation coppice as an effective technique for enhancing the extraction of several metals, among others Cd from the soil. However, Nissim et al. (2019) observed differences in Cd accumulation in poplars involved in short rotation management by individual size and age. Without the opportunity to study these factors in this analysis, we suppose that the latter points to a variable that should be considered in future assessment studies.

As individual studies are based on different experimental conditions, the synthetic findings of this paper should be defined in light of the potential effects of environmental influences. Besides the range of factors highlighted above in the cases of selected metals, factors such as study location (O’Connor et al. 2019), extreme abiotic stress (Sarma et al. 2021), and sampling season (Bidar et al. 2009) are of major importance when assessing metal accumulation in plant tissues. Thereby, it would be advantageous if future studies put more emphasis on the assessment of one or more of these factors, which would enable a more comprehensive overview done by integrating research built on their results.

### Correlation between soil pollution index (PI) and metal accumulation in *Populus* tissues

As we presented, there was no significant correlation between soil pollution intensity levels and metal (Cd, Cr, and Zn) uptake in plant parts of poplars, proposing that the accumulation of the metals was intensive from soils, independent of the degree of contamination in the growing media. Corresponding to metal accumulation alone, pollution intensity-dependent changes in tissue Cd, Cr, and Zn concentrations are also disparately discussed in papers.

In response to increasing metal stress, Jakovljević et al. (2014) found a non-significant but positive correlation in Cd concentration between soil and each of the studied plant parts (root, stem, and leaf) of *P. nigra*. The same conclusion has been drawn by Jun and Ling (2012) in cases of five poplar species; among plant parts, Cd enrichment was the most intensive in aboveground tissues. Radojčić Redovniković et al. (2017) reported on the limited applicability of *P. nigra* to an increased level of contamination. The authors found high remediation potential in soils with low to moderate contamination levels, while the correlation became nonlinear between Cd in tissues and soils by highly contaminated (Cd>25 mg kg^-1^) media. Under increased metal stress, it has been described that concentration drop in (primarily aboveground) tissues could be the result of the so-called dilution effect, which occurs when the intensity of biomass production of plants exceeds the extent of metal uptake (Robinson et al. 2000). In highly Cd-contaminated soils, not only changes in accumulation patterns but also toxicity symptoms such as growth reduction and necrosis have been observed (Marzilli et al. 2018), which could be the explanation behind our results linked to the occurrence of extreme (Cd>50 mg kg^-1^) soil concentrations in the papers involved.

The accumulation intensity of Cr was also influenced by changes in soil metal concentration. Chandra et al. (2016) indicated a non-significant positive correlation between soil and tissue concentrations in four poplar hybrids under different (5–500 mg l^-1^) contamination levels. In an earlier study, Renninger et al. (2013) reported an increase in *P. deltoides* leaf Cr concentration in response to elevated levels in soil; the correlation was also non-significant. Based on the nature of Cr in the soil-plant relation and according to the results presented here and in previous papers, we assume that poplars could make use of an effective exclusion mechanism when growing in highly and extremely contaminated soils (Cornejo et al. 2017), causing a decrease in Cr uptake intensity and translocation into stems and leaves.

Responses of poplars to Zn stress were also found to vary among the reported studies; however, the effect of the contamination level was considered to be major driving Zn uptake in earlier papers. In their abovementioned study, Renninger et al. (2013) found significantly higher Zn concentration in roots, stems, and leaves of *P. deltoides* from highly contaminated soils than in individuals from moderately and slightly contaminated ones. Testing two poplar clones, Fernàndez et al. (2012) demonstrated a uniform positive relationship between soil and tissue concentrations, with significant correlations for soil-young leaves, soil-stems in cases of both clones, and soil-roots in the case of one clone. In our paper, accumulation discrepancies due to toxicity triggered by high Zn contamination (in some cases soil >3000 mg kg^-1^) should not be excluded, while the role of metal interactions was also assumed (Gupta et al. 2016).

### Correlation between soil pH and metal accumulation in *Populus* tissues

We found a significant negative correlation between soil pH and leaf Mn concentration and a significant positive correlation between soil pH and stem Pb concentration of poplars. In the case of the other heavy metals discussed in this study (es. Zn, Cu), soil pH was not correlated with tissue-specific accumulation patterns.

Previous papers reported that a decrease in soil pH could result in higher mobility (bioavailability) of metals, which thus influences the amount accumulated by plants (Tan et al. 1997; Cloutier-Hurteau et al. 2010; Król et al. 2020). In the case of poplar-based remediation, a specific but not unique characteristic of poplars must also be taken into consideration; poplars can substantially alter acidity conditions in soils. Qasim et al. (2016) demonstrated that during the growth in a metal-contaminated environment, *P. euramericana* increased the pH of soil pore water by 0.3 to 0.6 units, which was explained by the anion and cation accumulation imbalance in roots. The relevant role of plants was also emphasized by Gonzaga et al. (2009), indicating a decrease in soil pH induced by root exudates. In a study on a 1-year-long experiment, Huynh et al. (2008) presented the contribution of poplars to the initial decrease and subsequent increase of soil pore water pH, thereby to the bioavailability of metals. In this paper, we found low accumulation rates and very wide, predominantly acidic pH ranges (2.7–7.3) for Mn. It is known that only a minor decrease in soil pH can substantially enrich the bioavailable Mn pool (Švec et al. 2016); thus, we assumed a similar trend behind the significant correlation indicated in our paper. A negative correlation was also found by Hu et al. (2019) for *P. hopeiensis* in soils contaminated by several metals. We found significant stem Pb accumulation under increasing alkalinity, which is not confirmed by previous papers (Heidari et al. 2005; Hu et al. 2019). In line with the transition of soil pH from acidic to alkaline ranges, Pb becomes even more immobile, exerting its toxic effects in a less pronounced manner on plant development; thereby physiological processes such as translocation mechanisms are more favored, enabling a relatively high degree of Pb flux into leaves (Brennan and Shelley 1999; Tangahu et al. 2011; Thakur et al. 2016). Concerning our results, the abovementioned mechanism could be, at least in part, the reason for the significant positive correlation between soil pH and Pb in the stem, as a major metal depository during the translocation process. The discrepancy in the results highlights the need for further research into the specific Pb uptake patterns in poplar.

### Temporal accumulation of metals in *Populus* tissues

For Cd in the stem, we found a significantly decreasing concentration with exposure time. In contrast, increased exposure time results in increased Cr in the stem and leaf, and for Mn in the stem, even until the 138th month. A great variety of results on different metals can also be seen in the relevant literature.

Highlighting their long-term applicability, Pierzynski et al. (2002) showed intensive Cd accumulation in poplar tissues throughout a 30-month-long experiment. Similarly, Scheid et al. (2009) indicated an increase in leaf litter concentration until the 25th month, suggesting the good translocation potential of Cd into the aboveground parts on a long-term basis. As we previously demonstrated, Cd tended to accumulate in the root and leaf, while the stem had relatively lower concentrations. Ruttens et al. (2011) reported that Cd accumulation intensity decreased in poplars after the second year of exposure, which could be related to the specific experimental condition. Temporal depletion of Cd in aboveground woody parts was observed earlier by Hu et al. (2019) in *P. hopeiensis*, who associated the phenomenon with the so-called stand age effect, suggesting a decline in uptake intensity in older (>10 years) trees than in younger ones. In addition, there is a high seasonal difference in the accumulation pattern of metals; Brekken and Steinnes (2004) identified a general seasonal decrease in Cd concentration in plant parts of poplars from spring to summer. Variations in experimental conditions in individual studies such as stand age and sampling time could have a major influence on the temporal decline of tissue Cd presented in this study.

In consonance with the results of this meta-analysis, Shukla et al. (2011) found a continuous increase in the accumulation of Cr in aboveground poplar tissues during a 12-month-long experiment. On a short-term (4-month long) scale, Zojaji et al. (2015) presented no major differences in Cr uptake in *P. deltoides*, assuming low short-term variation in Cr dynamics. Long-term effects were not reported consistently; Čudić et al. (2016) observed a significant decrease in Cr uptake in aboveground parts of poplars from the second to the third year of study, which was followed by an increase between the third and fifth years. In our study, Cr concentration of the stem and leaf significantly increased until the 138th month. Nevertheless, data were not available between the 49th and 137th months of experiments and, based on results from previous papers, potential major fluctuations in the intensity of Cr accumulation must not be excluded in this period.

Pottier et al. (2015) monitored the uptake of Mn in 14 poplar genotypes and observed an increase in leaf Mn concentration between April and November. Besides translocation among plant parts, long-term exposure to Mn stress could also facilitate intracellular sequestration mechanisms, which are effective tools for plants to cope with toxicity, and by which such compartmentation of Mn presented in this paper could be supported (Li et al. 2019). In contrast, Zárubová et al. (2015) found gradually decreasing Mn concentration during a 4-year-long study in aboveground parts of poplar clones. The authors attributed this pattern to the dilution effect. As mentioned above, we found low accumulation rates for Mn in poplars in contaminated soils compared to control ones. Yet, on a long (138 months) time scale, significant Mn accumulation occurred in poplar stem, which suggested the applicability of species in the long term (>10 years) rather than in the short- and mid-term (<5 years). It is worth noticing that similar to the results on Cr, continuous mid-and long-term accumulation intensity data are required to get a more extensive look at the Mn uptake patterns in poplars.

## Conclusions

In this paper, we demonstrated—with tools of meta-analysis—that poplars are widely applicable for phytoremediation purposes; however, there are major differences in the root, stem, and leaf accumulation among Cd, Cr, Cu, Mn, Ni, Pb, and Zn. Individuals growing in contaminated soils were able to accumulate Cd, Cr, Pb, and Zn in significantly higher concentrations than control individuals from uncontaminated soils. The uptake of Cu was partly significant, while that of Ni was non-significant in plant parts. Mn accumulation was generally very restricted. Poplars responded to the increasing pollution intensity levels of the growing media only with minor tissue concentration changes; except for Mn, significant accumulation was found for metals from low even to high pollution levels of the soils. In the stem, a decrease in soil pH values increased the accumulation of Mn, while it decreased the uptake of Pb. The accumulation of metals was also affected by exposure time; we observed a temporal decrease in stem Cd concentration, while stem and leaf Cr, and stem Mn accumulations became more intense with time, even until the 138th month of the experiment. Based on the results of this meta-analysis, we found poplars to be great candidates for the remediation of metal-contaminated soils; however, metal uptake preferences of the genus should be considered to achieve the most efficient remediation possible. Further, more extensive data on the factors influencing accumulation and long-term temporal uptake patterns would be needed to get a more thorough view of the prospects and limits of application.

## Supplementary information

ESM 1

## Data Availability

Research data and materials will be made available by request.
