# Supplementary material for: Heavy metal uptake by plant parts of Populus species: a meta-analysis
Source: Environ Sci Pollut Res Int. 2023 May 3;30(26):69416–30. doi: 10.1007/s11356-023-27244-2 (PMC10212865; doi:10.1007/s11356-023-27244-2)
Supplement: Supplementary file 1 — ESM 1 [file 11356_2023_27244_MOESM1_ESM.docx]

**Supplementary Materials A.** PRISMA flow diagram showing the selection process through the analysis (i.e., the number of studies identified, excluded, and included).

Records identified through
database searching
(n = 15,347)

## Screening

## Included

## Eligibility

## Identification

Additional records identified through other sources
(n = 0)

Records after duplicates removed
(n = 15,347)

Records screened
(n = 15,347)

Records excluded
(n = 14,194)

Full-text articles assessed for eligibility
(n = 1,153)

Full-text articles excluded, with reasons
(n = 973)

Studies included in qualitative synthesis
(n = 180)

Studies included in quantitative synthesis (meta-analysis)
(n = 29)

**Supplementary Materials B1.** Estimates and heterogeneities in the models for Cd.

| **Subgroup** | **Mean effect size (model)** | **Lower CI bound (model)** | **Upper CI bound (model)** | **SE (model)** | ***p* value (model)** | **Q (hetero-geneity)** | ***p* value (hetero-geneity)** | **I^2^** | **R^2^** |
| --- | --- | --- | --- | --- | --- | --- | --- | --- | --- |
| Leaf | -3.751 | -6.649 | -0.853 | 1.479 | 0.011 | 642.147 | <0.001 | 87% |  |
| Stem | -3.256 | -6.191 | -0.322 | 1.497 | 0.030 | 299.568 | <0.001 | 90% |  |
| Root | -4.131 | -7.044 | -1.219 | 1.486 | 0.005 | 334.284 | <0.001 | 89% |  |
| Overall | -4.524 | -5.065 | -3.984 | 0.276 | <0.001 | 1283.889 | <0.001 | 88% | 0% |

| **Component of variance** | ***Q*** | ***d.f.*** | ***p*** |
| --- | --- | --- | --- |
| Leaf | 642.147 | 85 | <0.001 |
| Stem | 299.568 | 30 | <0.001 |
| Root | 334.284 | 38 | <0.001 |
| Within | 1275.999 | 153 | <0.001 |
| Between | 2.107 | 2 | 0.349 |
| Total | 1283.889 | 155 | <0.001 |

**Supplementary Materials B2.** Estimates and heterogeneities in the models for Cr.

| **Subgroup** | **Mean effect size (model)** | **Lower CI bound (model)** | **Upper CI bound (model)** | **SE (model)** | ***p* value (model)** | **Q (hetero-geneity)** | ***p* value (hetero-geneity)** | **I^2^** | **R^2^** |
| --- | --- | --- | --- | --- | --- | --- | --- | --- | --- |
| Leaf | -2.786 | -5.488 | -0.084 | 1.378 | 0.043 | 47.330 | <0.001 | 75% |  |
| Stem | -2.894 | -5.662 | -0.126 | 1.412 | 0.040 | 31.700 | <0.001 | 84% |  |
| Root | -3.593 | -6.300 | -0.886 | 1.381 | 0.009 | 44.012 | <0.001 | 77% |  |
| Overall | -1.248 | -1.749 | -0.747 | 0.256 | <0.001 | 139.107 | <0.001 | 79% | 0% |

| **Component of variance** | ***Q*** | ***d.f.*** | ***p*** |
| --- | --- | --- | --- |
| Leaf | 47.330 | 12 | <0.001 |
| Stem | 31.700 | 5 | <0.001 |
| Root | 44.012 | 10 | <0.001 |
| Within | 123.042 | 27 | <0.001 |
| Between | 7.054 | 2 | 0.029 |
| Total | 139.107 | 29 | <0.001 |

**Supplementary Materials B3.** Estimates and heterogeneities in the models for Cu.

| **Subgroup** | **Mean effect size (model)** | **Lower CI bound (model)** | **Upper CI bound (model)** | **SE (model)** | ***p* value (model)** | **Q (hetero-geneity)** | ***p* value (hetero-geneity)** | **I^2^** | **R^2^** |
| --- | --- | --- | --- | --- | --- | --- | --- | --- | --- |
| Leaf | -1.405 | -2.699 | -0.110 | 0.661 | 0.033 | 299.281 | <0.001 | 83% |  |
| Stem | -1.278 | -2.594 | 0.038 | 0.672 | 0.057 | 157.433 | <0.001 | 82% |  |
| Root | -1.498 | -2.824 | -0.173 | 0.677 | 0.027 | 162.565 | <0.001 | 87% |  |
| Overall | -1.374 | -1.729 | -1.018 | 0.181 | <0.001 | 625.233 | <0.001 | 84% | 0% |

| **Component of variance** | ***Q*** | ***d.f.*** | ***p*** |
| --- | --- | --- | --- |
| Leaf | 299.281 | 52 | <0.001 |
| Stem | 157.433 | 28 | <0.001 |
| Root | 162.565 | 22 | <0.001 |
| Within | 619.280 | 101 | <0.001 |
| Between | 5.340 | 2 | 0.069 |
| Total | 625.233 | 103 | <0.001 |

**Supplementary Materials B4.** Estimates and heterogeneities in the models for Mn.

| **Subgroup** | **Mean effect size (model)** | **Lower CI bound (model)** | **Upper CI bound (model)** | **SE (model)** | ***p* value (model)** | **Q (hetero-geneity)** | ***p* value (hetero-geneity)** | **I^2^** | **R^2^** |
| --- | --- | --- | --- | --- | --- | --- | --- | --- | --- |
| Leaf | 1.106 | -0.439 | 2.651 | 0.788 | 0.161 | 99.815 | <0.001 | 81% |  |
| Stem | 1.059 | -0.576 | 2.694 | 0.834 | 0.204 | 35.630 | <0.001 | 83% |  |
| Overall | 0.941 | 0.246 | 1.636 | 0.355 | 0.008 | 137.316 | <0.001 | 81% | 0% |

| **Component of variance** | ***Q*** | ***d.f.*** | ***p*** |
| --- | --- | --- | --- |
| Leaf | 99.815 | 19 | <0.001 |
| Stem | 35.630 | 6 | <0.001 |
| Within | 135.444 | 25 | <0.001 |
| Between | 1.127 | 1 | 0.288 |
| Total | 137.316 | 26 | <0.001 |

**Supplementary Materials B5.** Estimates and heterogeneities in the models for Ni.

| **Subgroup** | **Mean effect size (model)** | **Lower CI bound (model)** | **Upper CI bound (model)** | **SE (model)** | ***p* value (model)** | **Q (hetero-geneity)** | ***p* value (hetero-geneity)** | **I^2^** | **R^2^** |
| --- | --- | --- | --- | --- | --- | --- | --- | --- | --- |
| Leaf | -0.850 | -2.010 | 0.310 | 0.592 | 0.151 | 42.346 | <0.001 | 79% |  |
| Stem | -0.406 | -1.734 | 0.923 | 0.678 | 0.994 | 20.557 | <0.001 | 85% |  |
| Root | -0.005 | -1.163 | 1.153 | 0.591 | 0.549 | 33.666 | <0.001 | 79% |  |
| Overall | -1.002 | -1.541 | -0.463 | 0.275 | <0.001 | 103.803 | <0.001 | 80% | 0% |

| **Component of variance** | ***Q*** | ***d.f.*** | ***p*** |
| --- | --- | --- | --- |
| Leaf | 42.346 | 9 | <0.001 |
| Stem | 20.557 | 3 | <0.001 |
| Root | 33.666 | 7 | <0.001 |
| Within | 96.569 | 19 | <0.001 |
| Between | 4.092 | 2 | 0.129 |
| Total | 103.803 | 21 | <0.001 |

**Supplementary Materials B6.** Estimates and heterogeneities in the models for Pb.

| **Subgroup** | **Mean effect size (model)** | **Lower CI bound (model)** | **Upper CI bound (model)** | **SE (model)** | ***p* value (model)** | **Q (hetero-geneity)** | ***p* value (hetero-geneity)** | **I^2^** | **R^2^** |
| --- | --- | --- | --- | --- | --- | --- | --- | --- | --- |
| Leaf | -3.177 | -6.390 | 0.004 | 1.639 | 0.053 | 119.248 | <0.001 | 77% |  |
| Stem | -2.672 | -5.936 | 0.593 | 1.666 | 0.109 | 75.388 | <0.001 | 87% |  |
| Root | -3.752 | -6.989 | -0.515 | 1.652 | 0.023 | 136.411 | <0.001 | 90% |  |
| Overall | -2.006 | -2.644 | -1.369 | 0.325 | <0.001 | 357.039 | <0.001 | 85% | 31.37% |

| **Component of variance** | ***Q*** | ***d.f.*** | ***p*** |
| --- | --- | --- | --- |
| Leaf | 119.248 | 27 | 0.019 |
| Stem | 75.388 | 10 | <0.001 |
| Root | 136.411 | 14 | <0.001 |
| Within | 331.047 | 51 | <0.001 |
| Between | 19.787 | 2 | <0.001 |
| Total | 357.039 | 53 | <0.001 |

**Supplementary Materials B7.** Estimates and heterogeneities in the models for Zn.

| **Subgroup** | **Mean effect size (model)** | **Lower CI bound (model)** | **Upper CI bound (model)** | **SE (model)** | ***p* value (model)** | **Q (hetero-geneity)** | ***p* value (hetero-geneity)** | **I^2^** | **R^2^** |
| --- | --- | --- | --- | --- | --- | --- | --- | --- | --- |
| Leaf | -3.125 | -4.524 | -1.726 | 0.714 | <0.001 | 512.401 | <0.001 | 85% |  |
| Stem | -2.094 | -3.516 | -0.672 | 0.726 | 0.004 | 132.243 | <0.001 | 77% |  |
| Root | -2.258 | -3.673 | -0.842 | 0.722 | 0.002 | 183.447 | <0.001 | 86% |  |
| Overall | -3.042 | -3.423 | -2.661 | 0.194 | <0.001 | 883.320 | <0.001 | 85% | 9.31% |

| **Component of variance** | ***Q*** | ***d.f.*** | ***p*** |
| --- | --- | --- | --- |
| Leaf | 512.401 | 75 | <0.001 |
| Stem | 132.243 | 30 | <0.001 |
| Root | 183.447 | 25 | <0.001 |
| Within | 828.092 | 130 | <0.001 |
| Between | 32.467 | 2 | <0.001 |
| Total | 883.320 | 132 | <0.001 |

**Supplementary Materials C1.** Funnel plots of the tested variables, regression tests for funnel plot asymmetry, and model results after trim and fill for Cd


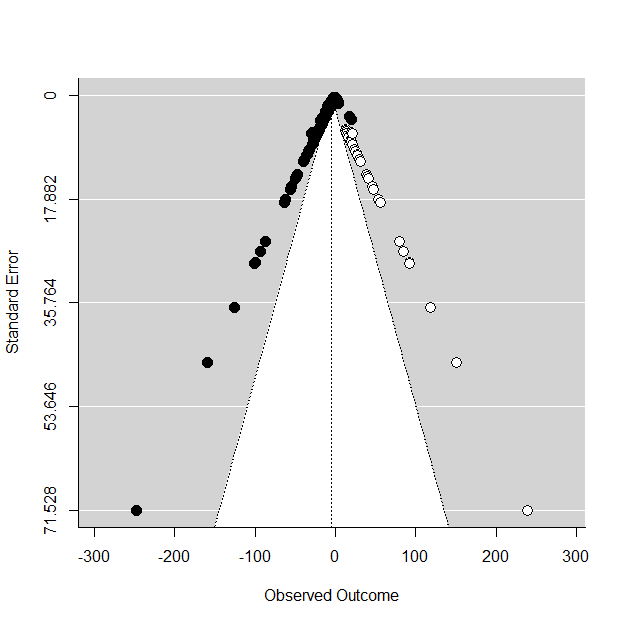


***Regression Test for Funnel Plot Asymmetry***

model: weighted regression with multiplicative dispersion

predictor: standard error

test for funnel plot asymmetry: t = -17.2597, d.f. = 154, p <0.0001

model: mixed-effects meta-regression model

predictor: standard error

test for funnel plot asymmetry: z = -23.0209, p <0.0001

***Model Results after Trim and Fill***:

Estimated number of missing studies on the right side: 36 (SE = 8.1676)

| logLik | Deviance | AIC | BIC | AICc | Q (hetero-geneity) | *p* value (hetero-geneity) | Tau^2^ | Tau | I^2^ | H^2^ |
| --- | --- | --- | --- | --- | --- | --- | --- | --- | --- | --- |
| -877.7110 | 1755.4219 | 1759.4219 | 1765.9265 | 1759.4858 | 1596.0152 | <0.0001 | 169.6590 | 13.0253 | 99.32% | 147.68 |

| Estimate Hedge's *g* | Lower bound | Upper bound | SE | z value | *p* value |
| --- | --- | --- | --- | --- | --- |
| -4.7003 | -6.7450 | -2.6556 | 1.0432 | -4.5055 | <0.0001 |

**Supplementary Materials C2.** Funnel plots of the tested variables, regression tests for funnel plot asymmetry, and model results after trim and fill for Cr


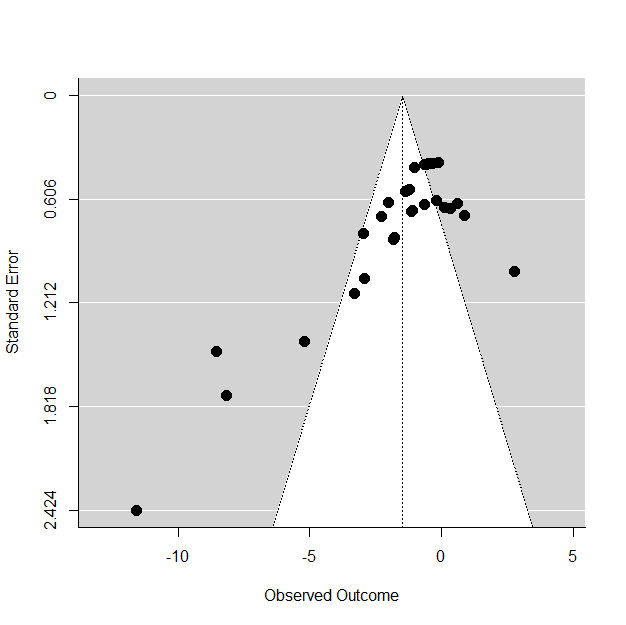


***Regression Test for Funnel Plot Asymmetry***

model: weighted regression with multiplicative dispersion

predictor: standard error

test for funnel plot asymmetry: t = -4.0857, d.f. = 28, p = 0.0003

model: mixed-effects meta-regression model

predictor: standard error

test for funnel plot asymmetry: z = -6.3471, p <0.0001

***Model Results after Trim and Fill***:

Estimated number of missing studies on the right side: 0 (SE = 2.9148)

| logLik | Deviance | AIC | BIC | AICc | Q (hetero-geneity) | *p* value (hetero-geneity) | Tau^2^ | Tau | I^2^ | H^2^ |
| --- | --- | --- | --- | --- | --- | --- | --- | --- | --- | --- |
| -68.4549 | 136.9098 | 140.9098 | 143.6444 | 141.3713 | 139.1069 | <0.0001 | 3.8230 | 1.9552 | 90.97% | 11.07 |

| Estimate Hedge's *g* | Lower bound | Upper bound | SE | z value | *p* value |
| --- | --- | --- | --- | --- | --- |
| -1.4722 | -2.2314 | -0.7129 | 0.3874 | -3.8004 | 0.0001 |

**Supplementary Materials C3.** Funnel plots of the tested variables, regression tests for funnel plot asymmetry, and model results after trim and fill for Cu


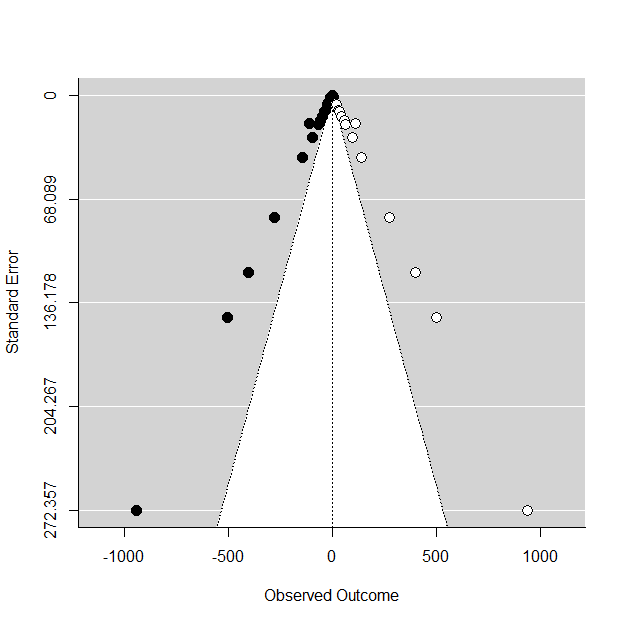


***Regression Test for Funnel Plot Asymmetry***

model: weighted regression with multiplicative dispersion

predictor: standard error

test for funnel plot asymmetry: t = -11.9338, d.f. = 102, p <0.0001

model: mixed-effects meta-regression model

predictor: standard error

test for funnel plot asymmetry: z = -17.4069, p <0.0001

***Model Results after Trim and Fill***:

Estimated number of missing studies on the right side: 13 (SE = 6.6136)

| logLik | Deviance | AIC | BIC | AICc | Q (hetero-geneity) | *p* value (hetero-geneity) | Tau^2^ | Tau | I^2^ | H^2^ |
| --- | --- | --- | --- | --- | --- | --- | --- | --- | --- | --- |
| -495.1542 | 990.3084 | 994.3084 | 999.8156 | 994.4146 | 794.7842 | <0.0001 | 3.6314 | 1.9056 | 88.11% | 8.41 |

| Estimate Hedge's *g* | Lower bound | Upper bound | SE | z value | *p* value |
| --- | --- | --- | --- | --- | --- |
| -1.4143 | -1.8460 | -0.9827 | 0.2203 | -6.4215 | <0.0001 |

**Supplementary Materials C4.** Funnel plots of the tested variables, regression tests for funnel plot asymmetry, and model results after trim and fill for Mn


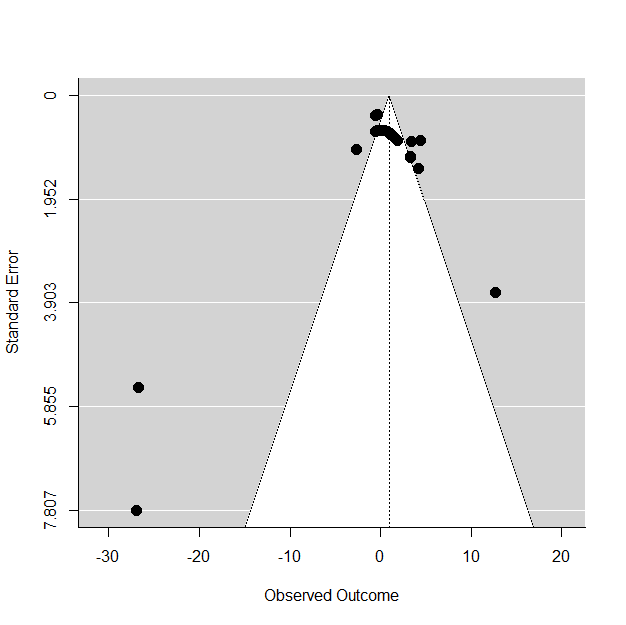


***Regression Test for Funnel Plot Asymmetry***

model: weighted regression with multiplicative dispersion

predictor: standard error

test for funnel plot asymmetry: t = 0.9530, d.f. = 25, p = 0.3497

model: mixed-effects meta-regression model

predictor: standard error

test for funnel plot asymmetry: z = -2.65883, p = 0.0096

***Model Results after Trim and Fill***:

Estimated number of missing studies on the right side: 0 (SE = 3.1632)

| logLik | Deviance | AIC | BIC | AICc | Q (hetero-geneity) | *p* value (hetero-geneity) | Tau^2^ | Tau | I^2^ | H^2^ |
| --- | --- | --- | --- | --- | --- | --- | --- | --- | --- | --- |
| -73.5657 | 147.1315 | 151.1315 | 153.6477 | 151.6532 | 137.3158 | <0.0001 | 2.1028 | 1.4501 | 79.83% | 4.96 |

| Estimate Hedge's *g* | Lower bound | Upper bound | SE | z value | *p* value |
| --- | --- | --- | --- | --- | --- |
| 0.9375 | 0.2834 | 1.5915 | 0.3337 | 2.8094 | 0.0050 |

**Supplementary Materials C5.** Funnel plots of the tested variables, regression tests for funnel plot asymmetry, and model results after trim and fill for Ni


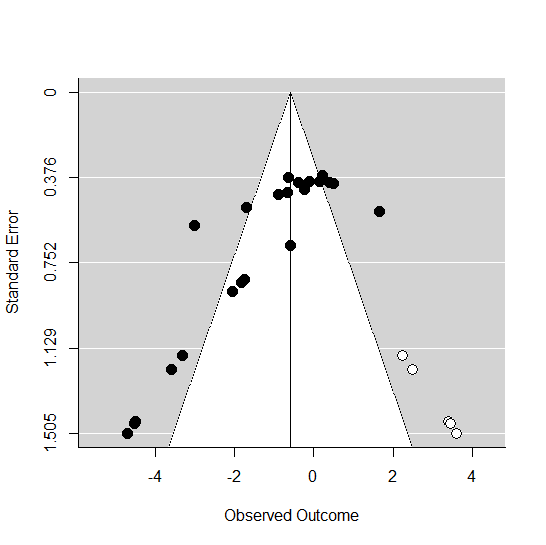


***Regression Test for Funnel Plot Asymmetry***

model: weighted regression with multiplicative dispersion

predictor: standard error

test for funnel plot asymmetry: t = -4.7971, d.f. = 20, p = 0.0001

model: mixed-effects meta-regression model

predictor: standard error

test for funnel plot asymmetry: z = -5.8387, p <0.0001

***Model Results after Trim and Fill***:

Estimated number of missing studies on the right side: 5 (SE = 3.1374)

| logLik | Deviance | AIC | BIC | AICc | Q (hetero-geneity) | *p* value (hetero-geneity) | Tau^2^ | Tau | I^2^ | H^2^ |
| --- | --- | --- | --- | --- | --- | --- | --- | --- | --- | --- |
| -57.5976 | 115.1952 | 119.1952 | 121.7113 | 119.7169 | 135.6173 | <0.0001 | 3.2101 | 1.7917 | 90.69% | 10.75 |

| Estimate Hedge's *g* | Lower bound | Upper bound | SE | z value | *p* value |
| --- | --- | --- | --- | --- | --- |
| -0.5908 | -1.3404 | 0.1589 | 0.3825 | -1.5445 | 0.1225 |

**Supplementary Materials C6.** Funnel plots of the tested variables, regression tests for funnel plot asymmetry, and model results after trim and fill for Pb


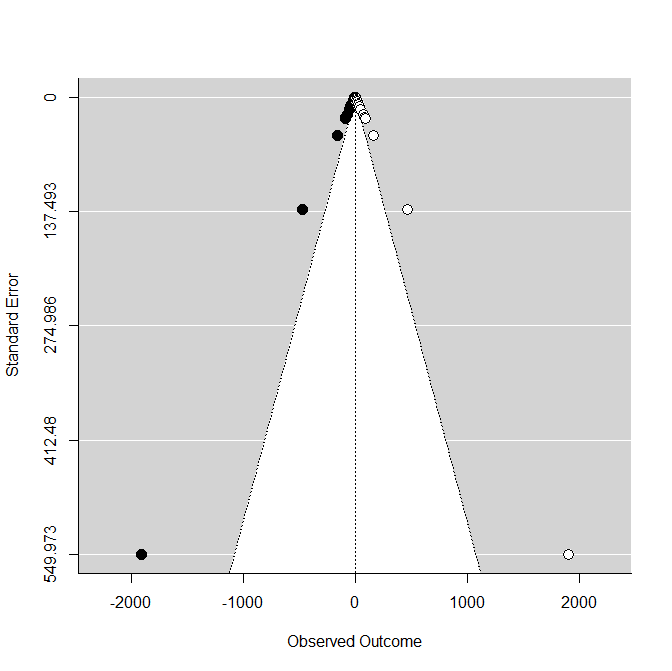


***Regression Test for Funnel Plot Asymmetry***

model: weighted regression with multiplicative dispersion

predictor: standard error

test for funnel plot asymmetry: t = -11.2078, d.f. = 52, p <0.0001

model: mixed-effects meta-regression model

predictor: standard error

test for funnel plot asymmetry: z = -15.8905, p <0.0001

***Model Results after Trim and Fill***:

Estimated number of missing studies on the right side: 19 (SE = 4.6265)

| logLik | Deviance | AIC | BIC | AICc | Q (hetero-geneity) | *p* value (hetero-geneity) | Tau^2^ | Tau | I^2^ | H^2^ |
| --- | --- | --- | --- | --- | --- | --- | --- | --- | --- | --- |
| -432.3176 | 568.1306 | 866.6351 | 868.9256 | 866.6914 | 568.1306 | <0.0001 | 6.4959 | 2.5487 | 87.33% | 7.89 |

| Estimate Hedge's *g* | Lower bound | Upper bound | SE | z value | *p* value |
| --- | --- | --- | --- | --- | --- |
| -0.6820 | -0.8792 | -0.4849 | 0.1006 | -6.7808 | <0.0001 |

**Supplementary Materials C7.** Funnel plots of the tested variables, regression tests for funnel plot asymmetry, and model results after trim and fill for Zn


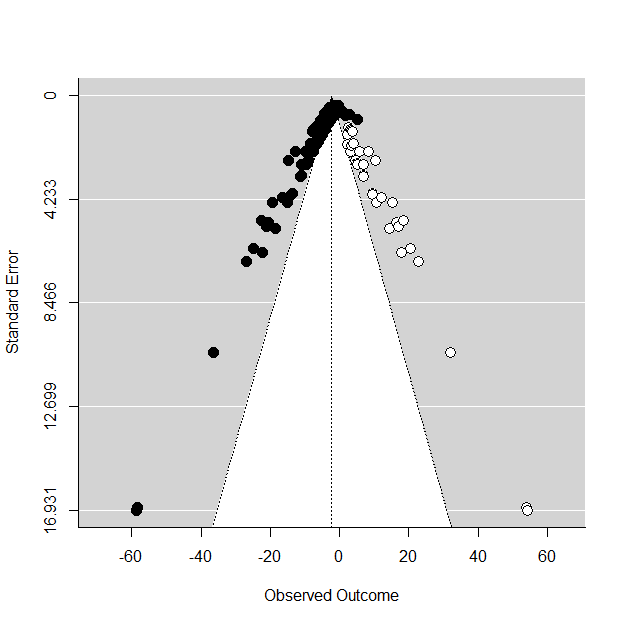


***Regression Test for Funnel Plot Asymmetry***

model: weighted regression with multiplicative dispersion

predictor: standard error

test for funnel plot asymmetry: t = -12.8243, d.f. = 131, p <0.0001

model: mixed-effects meta-regression model

predictor: standard error

test for funnel plot asymmetry: z = -18.0224, p <0.0001

***Model Results after Trim and Fill***:

Estimated number of missing studies on the right side: 34 (SE = 7.5084)

| logLik | Deviance | AIC | BIC | AICc | Q (hetero-geneity) | *p* value (hetero-geneity) | Tau^2^ | Tau | I^2^ | H^2^ |
| --- | --- | --- | --- | --- | --- | --- | --- | --- | --- | --- |
| -580.8033 | 1161.6065 | 1165.6065 | 1171.8305 | 1165.6801 | 1232.4093 | <0.0001 | 26.0305 | 5.1020 | 97.08% | 34.28 |

| Estimate Hedge's *g* | Lower bound | Upper bound | SE | z value | *p* value |
| --- | --- | --- | --- | --- | --- |
| -2.2427 | -3.0859 | -1.3995 | 0.4302 | -5.2131 | <0.0001 |

**Supplementary Materials D.** Linear regressions between soil pollution intensity and effect size.

***Cd-Leaf***

| **Component of variance** | ***d.f.*** | **Sum of squares** | **Mean square** | **F value** | ***p* value** |
| --- | --- | --- | --- | --- | --- |
| Model | 1 | 107.4936 | 107.494 | 1.024 | 0.317 |
| Error | 50 | 5250.728 | 105.015 |  |  |
| Total | 51 | 5358.222 |  |  |  |

***Cr-Root***

| **Component of variance** | ***d.f.*** | **Sum of squares** | **Mean square** | **F value** | ***p* value** |
| --- | --- | --- | --- | --- | --- |
| Model | 1 | 9.040 | 9.040 | 1.028 | 0.368 |
| Error | 4 | 35.166 | 8.792 |  |  |
| Total | 5 | 44.206 |  |  |  |

***Zn-Stem***

| **Component of variance** | ***d.f.*** | **Sum of squares** | **Mean square** | **F value** | ***p* value** |
| --- | --- | --- | --- | --- | --- |
| Model | 1 | 13.948 | 13.948 | 0.415 | 0.526 |
| Error | 22 | 740.088 | 33.640 |  |  |
| Total | 23 | 754.036 |  |  |  |

***Zn-Leaf***

| **Component of variance** | ***d.f.*** | **Sum of squares** | **Mean square** | **F value** | ***p* value** |
| --- | --- | --- | --- | --- | --- |
| Model | 1 | 29.805 | 29.805 | 0.409 | 0.525 |
| Error | 63 | 4592.630 | 72.899 |  |  |
| Total | 64 | 4622.435 |  |  |  |

**Supplementary Materials E1.** Linear regressions between soil pH and effect size for Cd

***Cd-leaf***

| **Component of variance** | ***d.f.*** | **Sum of squares** | **Mean square** | **F value** | ***p* value** |
| --- | --- | --- | --- | --- | --- |
| Model | 1 | 824.563 | 824.563 | 1.937 | 0.168 |
| Error | 80 | 34059.269 | 425.741 |  |  |
| Total | 81 | 34883.833 |  |  |  |

***Cd-stem***

| **Component of variance** | ***d.f.*** | **Sum of squares** | **Mean square** | **F value** | ***p* value** |
| --- | --- | --- | --- | --- | --- |
| Model | 1 | 208.410 | 208.410 | 0.638 | 0.431 |
| Error | 29 | 9469.460 | 326.533 |  |  |
| Total | 30 | 9677.871 |  |  |  |

***Cd-root***

| **Component of variance** | ***d.f.*** | **Sum of squares** | **Mean square** | **F value** | ***p* value** |
| --- | --- | --- | --- | --- | --- |
| Model | 1 | 2984.310 | 2984.310 | 1.163 | 0.288 |
| Error | 35 | 89810.909 | 2566.026 |  |  |
| Total | 36 | 92795.219 |  |  |  |

**Supplementary Materials E2.** Linear regressions between soil pH and effect size for Cr

***Cr-leaf***

| **Component of variance** | ***d.f.*** | **Sum of squares** | **Mean square** | **F value** | ***p* value** |
| --- | --- | --- | --- | --- | --- |
| Model | 1 | 2.012 | 2.012 | 0.198 | 0.670 |
| Error | 7 | 71.116 | 10.159 |  |  |
| Total | 8 | 73.128 |  |  |  |

***Cr-stem***

| **Component of variance** | ***d.f.*** | **Sum of squares** | **Mean square** | **F value** | ***p* value** |
| --- | --- | --- | --- | --- | --- |
| Model | 1 | 40.105 | 40.105 | 2.372 | 0.198 |
| Error | 4 | 67.620 | 16.905 |  |  |
| Total | 5 | 107.725 |  |  |  |

***Cr-root***

| **Component of variance** | ***d.f.*** | **Sum of squares** | **Mean square** | **F value** | ***p* value** |
| --- | --- | --- | --- | --- | --- |
| Model | 1 | 11.095 | 11.095 | 2.089 | 0.192 |
| Error | 7 | 37.175 | 5.311 |  |  |
| Total | 8 | 48.270 |  |  |  |

**Supplementary Materials E3.** Linear regressions between soil pH and effect size for Cu

***Cu-leaf***

| **Component of variance** | ***d.f.*** | **Sum of squares** | **Mean square** | **F value** | ***p* value** |
| --- | --- | --- | --- | --- | --- |
| Model | 1 | 10024.048 | 10024.048 | 1.774 | 0.190 |
| Error | 44 | 248578.816 | 5649.519 |  |  |
| Total | 45 | 258602.864 |  |  |  |

***Cu-stem***

| **Component of variance** | ***d.f.*** | **Sum of squares** | **Mean square** | **F value** | ***p* value** |
| --- | --- | --- | --- | --- | --- |
| Model | 1 | 8174.195 | 8174.195 | 1.450 | 0.239 |
| Error | 26 | 146537.925 | 5636.074 |  |  |
| Total | 27 | 154712.120 |  |  |  |

***Cu-root***

| **Component of variance** | ***d.f.*** | **Sum of squares** | **Mean square** | **F value** | ***p* value** |
| --- | --- | --- | --- | --- | --- |
| Model | 1 | 49832.422 | 49832.422 | 1.041 | 0.322 |
| Error | 17 | 814132.314 | 47890.136 |  |  |
| Total | 18 | 863964.736 |  |  |  |

**Supplementary Materials E4.** Linear regressions between soil pH and effect size for Mn

***Mn-leaf***

| **Component of variance** | ***d.f.*** | **Sum of squares** | **Mean square** | **F value** | ***p* value** |
| --- | --- | --- | --- | --- | --- |
| Model | 1 | 187.727 | 187.727 | 4.320 | 0.052 |
| Error | 18 | 782.110 | 43.451 |  |  |
| Total | 19 | 969.837 |  |  |  |

***Mn-stem***

| **Component of variance** | ***d.f.*** | **Sum of squares** | **Mean square** | **F value** | ***p* value** |
| --- | --- | --- | --- | --- | --- |
| Model | 1 | 135.187 | 135.187 | 1.303 | 0.305 |
| Error | 5 | 518.639 | 103.728 |  |  |
| Total | 6 | 653.827 |  |  |  |

**Supplementary Materials E5.** Linear regressions between soil pH and effect size for Ni

***Ni-leaf***

| **Component of variance** | ***d.f.*** | **Sum of squares** | **Mean square** | **F value** | ***p* value** |
| --- | --- | --- | --- | --- | --- |
| Model | 1 | 2.410 | 2.410 | 0.594 | 0.484 |
| Error | 4 | 16.238 | 4.060 |  |  |
| Total | 5 | 18.648 |  |  |  |

***Ni-stem***

| **Component of variance** | ***d.f.*** | **Sum of squares** | **Mean square** | **F value** | ***p* value** |
| --- | --- | --- | --- | --- | --- |
| Model | 1 | 9.564 | 9.564 | 5.426 | 0.145 |
| Error | 2 | 3.525 | 1.763 |  |  |
| Total | 3 | 13.090 |  |  |  |

***Ni-root***

| **Component of variance** | ***d.f.*** | **Sum of squares** | **Mean square** | **F value** | ***p* value** |
| --- | --- | --- | --- | --- | --- |
| Model | 1 | 3.861 | 3.861 | 2.947 | 0.161 |
| Error | 4 | 5.241 | 1.310 |  |  |
| Total | 5 | 9.102 |  |  |  |

**Supplementary Materials E6.** Linear regressions between soil pH and effect size for Pb

***Pb-leaf***

| **Component of variance** | ***d.f.*** | **Sum of squares** | **Mean square** | **F value** | ***p* value** |
| --- | --- | --- | --- | --- | --- |
| Model | 1 | 117134.290 | 117134.290 | 0.770 | 0.390 |
| Error | 22 | 3.34674E6 | 152124.686 |  |  |
| Total | 23 | 3.46388E6 |  |  |  |

***Pb-stem***

| **Component of variance** | ***d.f.*** | **Sum of squares** | **Mean square** | **F value** | ***p* value** |
| --- | --- | --- | --- | --- | --- |
| Model | 1 | 10960.302 | 10960.302 | 6.362 | 0.0327 |
| Error | 9 | 15505.020 | 1722.780 |  |  |
| Total | 10 | 26465.323 |  |  |  |

***Pb-root***

| **Component of variance** | ***d.f.*** | **Sum of squares** | **Mean square** | **F value** | ***p* value** |
| --- | --- | --- | --- | --- | --- |
| Model | 1 | 17440.840 | 17440.840 | 1.112 | 0.314 |
| Error | 11 | 172461.112 | 15678.283 |  |  |
| Total | 12 | 189901.952 |  |  |  |

**Supplementary Materials E7.** Linear regressions between soil pH and effect size for Zn

***Zn-leaf***

| **Component of variance** | ***d.f.*** | **Sum of squares** | **Mean square** | **F value** | ***p* value** |
| --- | --- | --- | --- | --- | --- |
| Model | 1 | 51.812 | 51.812 | 0.419 | 0.520 |
| Error | 67 | 8283.747 | 123.638 |  |  |
| Total | 68 | 8335.558 |  |  |  |

***Zn-stem***

| **Component of variance** | ***d.f.*** | **Sum of squares** | **Mean square** | **F value** | ***p* value** |
| --- | --- | --- | --- | --- | --- |
| Model | 1 | 1.4311 | 1.4311 | 0.0465 | 0.831 |
| Error | 28 | 861.546 | 30.759 |  |  |
| Total | 29 | 862.977 |  |  |  |

***Zn-root***

| **Component of variance** | ***d.f.*** | **Sum of squares** | **Mean square** | **F value** | ***p* value** |
| --- | --- | --- | --- | --- | --- |
| Model | 1 | 4.158 | 4.158 | 0.081 | 0.778 |
| Error | 21 | 1073.288 | 51.109 |  |  |
| Total | 22 | 1077.445 |  |  |  |

**Supplementary Materials F1.** Linear regressions between exposure time and effect size for Cd

***Cd-leaf***

| **Component of variance** | ***d.f.*** | **Sum of squares** | **Mean square** | **F value** | ***p* value** |
| --- | --- | --- | --- | --- | --- |
| Model | 1 | 371.405 | 371.405 | 0.872 | 0.353 |
| Error | 82 | 34910.988 | 425.744 |  |  |
| Total | 83 | 35282.393 |  |  |  |

***Cd-root***

| **Component of variance** | ***d.f.*** | **Sum of squares** | **Mean square** | **F value** | ***p* value** |
| --- | --- | --- | --- | --- | --- |
| Model | 1 | 2860.007 | 2860.007 | 1.157 | 0.289 |
| Error | 37 | 91437.150 | 2471.274 |  |  |
| Total | 38 | 94297.156 |  |  |  |

**Supplementary Materials F2.** Linear regressions between exposure time and effect size for Cr

***Cr-root***

| **Component of variance** | ***d.f.*** | **Sum of squares** | **Mean square** | **F value** | ***p* value** |
| --- | --- | --- | --- | --- | --- |
| Model | 1 | 3.225 | 3.225 | 0.543 | 0.480 |
| Error | 9 | 53.441 | 5.938 |  |  |
| Total | 10 | 56.666 |  |  |  |

**Supplementary Materials F3.** Linear regressions between exposure time and effect size for Cu

***Cu-leaf***

| **Component of variance** | ***d.f.*** | **Sum of squares** | **Mean square** | **F value** | ***p* value** |
| --- | --- | --- | --- | --- | --- |
| Model | 1 | 453.276 | 453.276 | 0.086 | 0.771 |
| Error | 49 | 259066.951 | 5287.081 |  |  |
| Total | 50 | 259520.227 |  |  |  |

***Cu-stem***

| **Component of variance** | ***d.f.*** | **Sum of squares** | **Mean square** | **F value** | ***p* value** |
| --- | --- | --- | --- | --- | --- |
| Model | 1 | 898.300 | 898.300 | 0.157 | 0.695 |
| Error | 27 | 154028.822 | 5704.771 |  |  |
| Total | 28 | 154927.122 |  |  |  |

***Cu-root***

| **Component of variance** | ***d.f.*** | **Sum of squares** | **Mean square** | **F value** | ***p* value** |
| --- | --- | --- | --- | --- | --- |
| Model | 1 | 17102.194 | 17102.194 | 0.395 | 0.537 |
| Error | 20 | 865781.018 | 702.610 |  |  |
| Total | 21 | 882883.212 |  |  |  |

**Supplementary Materials F4.** Linear regressions between exposure time and effect size for Mn

***Mn-leaf***

| **Component of variance** | ***d.f.*** | **Sum of squares** | **Mean square** | **F value** | ***p* value** |
| --- | --- | --- | --- | --- | --- |
| Model | 1 | 24.174 | 24.174 | 0.414 | 0.529 |
| Error | 16 | 934.352 | 58.397 |  |  |
| Total | 17 | 958.526 |  |  |  |

**Supplementary Materials F5.** Linear regressions between exposure time and effect size for Ni

***Ni-leaf***

| **Component of variance** | ***d.f.*** | **Sum of squares** | **Mean square** | **F value** | ***p* value** |
| --- | --- | --- | --- | --- | --- |
| Model | 1 | 4.179 | 4.179 | 1.227 | 0.300 |
| Error | 8 | 27.255 | 3.407 |  |  |
| Total | 9 | 31.434 |  |  |  |

***Ni-stem***

| **Component of variance** | ***d.f.*** | **Sum of squares** | **Mean square** | **F value** | ***p* value** |
| --- | --- | --- | --- | --- | --- |
| Model | 1 | 9.564 | 9.564 | 5.426 | 0.145 |
| Error | 2 | 3.525 | 1.763 |  |  |
| Total | 3 | 13.089 |  |  |  |

***Ni-root***

| **Component of variance** | ***d.f.*** | **Sum of squares** | **Mean square** | **F value** | ***p* value** |
| --- | --- | --- | --- | --- | --- |
| Model | 1 | 0.376 | 0.376 | 0.208 | 0.665 |
| Error | 6 | 10.843 | 1.807 |  |  |
| Total | 7 | 11.219 |  |  |  |

**Supplementary Materials F6.** Linear regressions between exposure time and effect size for Pb

***Pb-leaf***

| **Component of variance** | ***d.f.*** | **Sum of squares** | **Mean square** | **F value** | ***p* value** |
| --- | --- | --- | --- | --- | --- |
| Model | 1 | 15660.626 | 15660.626 | 0.109 | 0.745 |
| Error | 24 | 3.46062E6 | 144192.349 |  |  |
| Total | 25 | 3.47628E6 |  |  |  |

***Pb-stem***

| **Component of variance** | ***d.f.*** | **Sum of squares** | **Mean square** | **F value** | ***p* value** |
| --- | --- | --- | --- | --- | --- |
| Model | 1 | 3.326 | 3.326 | 0.001 | 0.974 |
| Error | 9 | 26461.997 | 2940.222 |  |  |
| Total | 10 | 26465.323 |  |  |  |

***Pb-root***

| **Component of variance** | ***d.f.*** | **Sum of squares** | **Mean square** | **F value** | ***p* value** |
| --- | --- | --- | --- | --- | --- |
| Model | 1 | 3354.344 | 3354.344 | 0.226 | 0.642 |
| Error | 13 | 192662.826 | 14820.217 |  |  |
| Total | 14 | 196017.170 |  |  |  |

**Supplementary Materials F7.** Linear regressions between exposure time and effect size for Zn

***Zn-leaf***

| **Component of variance** | ***d.f.*** | **Sum of squares** | **Mean square** | **F value** | ***p* value** |
| --- | --- | --- | --- | --- | --- |
| Model | 1 | 31.814 | 31.814 | 0.270 | 0.605 |
| Error | 72 | 8473.165 | 117.683 |  |  |
| Total | 73 | 8504.979 |  |  |  |

***Zn-stem***

| **Component of variance** | ***d.f.*** | **Sum of squares** | **Mean square** | **F value** | ***p* value** |
| --- | --- | --- | --- | --- | --- |
| Model | 1 | 0.131 | 0.131 | 0.004 | 0.948 |
| Error | 29 | 865.241 | 29.836 |  |  |
| Total | 30 | 865.372 |  |  |  |

***Zn-root***

| **Component of variance** | ***d.f.*** | **Sum of squares** | **Mean square** | **F value** | ***p* value** |
| --- | --- | --- | --- | --- | --- |
| Model | 1 | 77.485 | 77.485 | 1.785 | 0.194 |
| Error | 24 | 1041.568 | 43.399 |  |  |
| Total | 25 | 1119.053 |  |  |  |
